# *trans*-Dibromidobis(triphenyl­phosphane)platinum(II) chloro­form monosolvate

**DOI:** 10.1107/S1600536811016849

**Published:** 2011-05-07

**Authors:** Alexandra M. Z. Slawin, Paul G. Waddell, J. Derek Woollins

**Affiliations:** a Department of Chemistry, University of St Andrews, St Andrews KY16 9ST, Scotland; b Cavendish Laboratory, University of Cambridge, J. J. Thomson Avenue, Cambridge CB3 0HE, England

## Abstract

Both the platininum complex and the solvent mol­ecule of the title compound, [PtBr_2_(C_18_H_15_P)_2_]·CHCl_3_, are located on a twofold rotation axis. The CH unit and the Cl atoms of the CHCl_3_ mol­ecule are disordered over two equally occupied positions. The complex shows a *trans* square-planar geometry about the Pt atom.

## Related literature

For the dichloro­methane solvate analogue of the title structure, see: Sharma *et al.* (2003 ▶). For the structure of the *cis* isomer of the title complex, see: Rigamonti *et al.* (2010 ▶). For the low temperature structure of the chloro­form solvate of the *cis* isomer of the title complex, see: Waddell *et al.* (2010 ▶). For more information on the effect of the *trans* influence of ligands on platinum-phospho­rus complexes, see: Allen *et al.* (1970 ▶); Appleton *et al.* (1973 ▶).
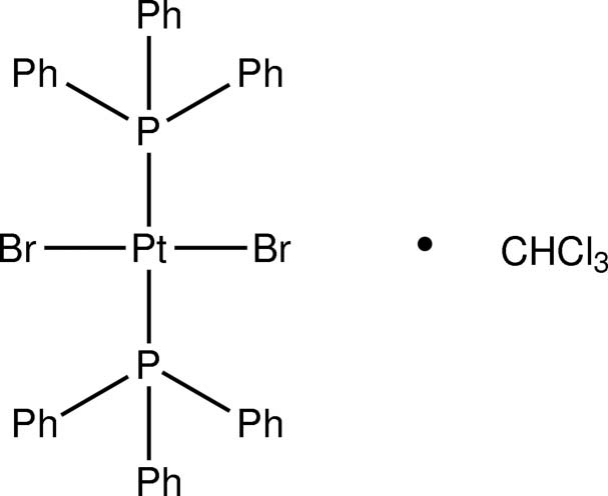


## Experimental

### 

#### Crystal data

[PtBr_2_(C_18_H_15_P)_2_]·CHCl_3_
*M* *_r_* = 998.82
Monoclinic, 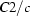

*a* = 12.2581 (11) Å
*b* = 14.5375 (13) Å
*c* = 20.1433 (18) Å
β = 92.402 (6)°
*V* = 3586.4 (6) Å^3^
*Z* = 4
Mo *K*α radiation
μ = 6.48 mm^−1^
*T* = 125 K
0.20 × 0.12 × 0.09 mm

#### Data collection

Rigaku SCXmini diffractometer
Absorption correction: multi-scan (*ABSCOR*; Higashi, 1995 ▶) *T*_min_ = 0.364, *T*_max_ = 0.600
14789 measured reflections
3161 independent reflections
2495 reflections with *I* > 2σ(*I*)
*R*_int_ = 0.043

#### Refinement

*R*[*F* ^2^ > 2σ(*F* ^2^)] = 0.028
*wR*(*F* ^2^) = 0.047
*S* = 1.09
3161 reflections
218 parameters
H atoms treated by a mixture of independent and constrained refinement
Δρ_max_ = 0.58 e Å^−3^
Δρ_min_ = −0.71 e Å^−3^

### 

Data collection: *SCXmini Benchtop Crystallography System Software* (Rigaku, 2006*b* ▶); cell refinement: *PROCESS-AUTO* (Rigaku, 1998 ▶); data reduction: *PROCESS-AUTO*; program(s) used to solve structure: *SHELXS97* (Sheldrick, 2008 ▶); program(s) used to refine structure: *SHELXL97* (Sheldrick, 2008 ▶); molecular graphics: *Crystal­Structure* (Rigaku, 2006*a* ▶); software used to prepare material for publication: *CrystalStructure*.

## Supplementary Material

Crystal structure: contains datablocks global, I. DOI: 10.1107/S1600536811016849/bt5541sup1.cif

Structure factors: contains datablocks I. DOI: 10.1107/S1600536811016849/bt5541Isup2.hkl

Additional supplementary materials:  crystallographic information; 3D view; checkCIF report

## Figures and Tables

**Fig. 1.**
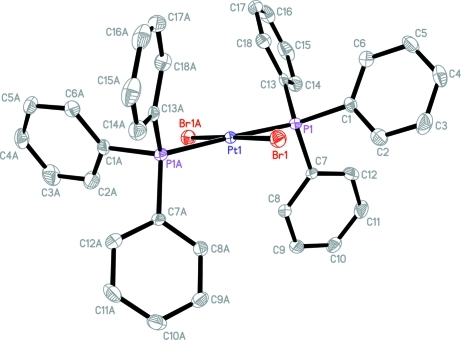
The structure of the title compound with displacement ellipsoids drawn at the 50% probability level, hydrogen atoms and the disordered CHCl3 omitted for clarity. Symmetry operator for generating equivalent atoms (A): -x + 1,y,-z + 1/2.

## supplementary crystallographic information

### Comment

The *trans*-Bis(triphenylphosphane)dibromoplatinum(II) molecule in the title structure bears a close resemblance to that of the dichloromethane solvate of the same complex (Sharma *et al.* 2003). The geometry about platinum is similar in both structures. As would be expected, due to the different *trans* infuences of triphenylphosphane and bromide (Allen *et al.* 1970; Appleton *et al.* 1973), the Pt—Br distances are observed to be shorter and the Pt—P distances longer in the title structure than those of the structures of the *cis* isomer of the complex (Rigamonti *et al.* 2010; Waddell *et al.* 2010). A twofold disorder is observed in the chloroform molecule.

### Experimental

*trans*-bis(benzonitrile)platinum(II) dibromide (0.5 g, 0.9 mmol) was vigorously stirred in acetone (20 ml), to which triphenylphosphane (0.472 g, 1.8 mmol) dissolved in acetone (20 ml) was added, affording a yellow precipitate. Crystals were grown for X-ray crystallography *via* slow diffusion of hexane into a solution of the product in chloroform. Yield: 0.726 g (0.8 mmol), 92%.

### Refinement

All H atoms   were included in calculated positions (C—H distances are 0.96 Å for methyl H atoms, 0.97 Å for methylene H atoms and 0.98 Å for methine H atoms) and were refined as riding atoms with *U*_iso_(H) = 1.2 *U*_eq_(parent atom, methylene and methine H atoms) or *U*_iso_(H) = 1.5 *U*_eq_ (parent atom, methyl H atoms).

### Crystal data

**Table:**

[PtBr_2_(C_18_H_15_P)_2_]·CHCl_3_	*F*(000) = 1928
*M**_r_* = 998.82	*D*_x_ = 1.85 Mg m^−^^3^
Monoclinic, *C*2/*c*	Mo *K*α radiation, λ = 0.71075 Å
Hall symbol: -C 2yc	Cell parameters from 14699 reflections
*a* = 12.2581 (11) Å	θ = 3–27.4°
*b* = 14.5375 (13) Å	µ = 6.48 mm^−^^1^
*c* = 20.1433 (18) Å	*T* = 125 K
β = 92.402 (6)°	Prism, yellow
*V* = 3586.4 (6) Å^3^	0.2 × 0.12 × 0.09 mm
*Z* = 4	

### Data collection

**Table:**

Rigaku SCXmini diffractometer	3161 independent reflections
graphite	2495 reflections with *I* > 2σ(*I*)
Detector resolution: 6.85 pixels mm^-1^	*R*_int_ = 0.043
ω scans	θ_max_ = 25°, θ_min_ = 3.0°
Absorption correction: multi-scan (*ABSCOR*; Higashi, 1995)	*h* = −14→14
*T*_min_ = 0.364, *T*_max_ = 0.600	*k* = −17→17
14789 measured reflections	*l* = −23→23

### Refinement

**Table:**

Refinement on *F*^2^	Primary atom site location: structure-invariant direct methods
Least-squares matrix: full	Secondary atom site location: difference Fourier map
*R*[*F*^2^ > 2σ(*F*^2^)] = 0.028	Hydrogen site location: inferred from neighbouring sites
*wR*(*F*^2^) = 0.047	H atoms treated by a mixture of independent and constrained refinement
*S* = 1.09	*w* = 1/[σ^2^(*F*_o_^2^) + (0.0095*P*)^2^ + 13.9423*P*] where *P* = (*F*_o_^2^ + 2*F*_c_^2^)/3
3161 reflections	(Δ/σ)_max_ = 0.001
218 parameters	Δρ_max_ = 0.58 e Å^−^^3^
0 restraints	Δρ_min_ = −0.71 e Å^−^^3^

### Special details

**Table:**

Geometry. All s.u.'s (except the s.u. in the dihedral angle between two l.s. planes) are estimated using the full covariance matrix. The cell s.u.'s are taken into account individually in the estimation of s.u.'s in distances, angles and torsion angles; correlations between s.u.'s in cell parameters are only used when they are defined by crystal symmetry. An approximate (isotropic) treatment of cell s.u.'s is used for estimating s.u.'s involving l.s. planes.
Refinement. Refinement of *F*^2^ against ALL reflections. The weighted *R*-factor *wR* and goodness of fit *S* are based on *F*^2^, conventional *R*-factors *R* are based on *F*, with *F* set to zero for negative *F*^2^. The threshold expression of *F*^2^ > 2σ(*F*^2^) is used only for calculating *R*-factors(gt) *etc*. and is not relevant to the choice of reflections for refinement. *R*-factors based on *F*^2^ are statistically about twice as large as those based on *F*, and *R*- factors based on ALL data will be even larger.

### Fractional atomic coordinates and isotropic or equivalent isotropic displacement parameters (Å^2^)

**Table:**

	*x*	*y*	*z*	*U*_iso_*/*U*_eq_	Occ. (<1)
Pt1	0.5	0.247997 (17)	0.25	0.01380 (6)	
Br1	0.67746 (3)	0.24647 (3)	0.199294 (18)	0.02189 (10)	
Cl1	0.06034 (12)	0.09593 (10)	0.19017 (7)	0.0479 (3)	
Cl2	−0.0238 (3)	0.26606 (18)	0.2359 (2)	0.0593 (12)	0.5
P1	0.40680 (8)	0.24595 (8)	0.14711 (5)	0.0152 (2)	
C1	0.4819 (3)	0.2713 (2)	0.07256 (19)	0.0166 (9)	
C2	0.5531 (4)	0.2057 (3)	0.0482 (2)	0.0276 (11)	
H2	0.5621	0.1485	0.0706	0.033*	
C3	0.6107 (4)	0.2230 (3)	−0.0081 (2)	0.0330 (12)	
H3	0.6581	0.1774	−0.0245	0.04*	
C4	0.5996 (4)	0.3062 (3)	−0.0405 (2)	0.0276 (11)	
H4	0.6405	0.3183	−0.0786	0.033*	
C5	0.5295 (4)	0.3713 (3)	−0.0178 (2)	0.0274 (11)	
H5	0.5203	0.428	−0.0408	0.033*	
C6	0.4719 (3)	0.3544 (3)	0.0389 (2)	0.0231 (10)	
H6	0.4249	0.4006	0.0549	0.028*	
C7	0.3475 (3)	0.1333 (2)	0.1283 (2)	0.0153 (9)	
C8	0.3484 (3)	0.0654 (3)	0.1772 (2)	0.0181 (10)	
H8	0.381	0.0775	0.2199	0.022*	
C9	0.3014 (4)	−0.0204 (3)	0.1635 (2)	0.0246 (10)	
H9	0.3021	−0.0664	0.1969	0.03*	
C10	0.2541 (4)	−0.0387 (3)	0.1017 (2)	0.0243 (10)	
H10	0.22	−0.0964	0.0932	0.029*	
C11	0.2563 (4)	0.0275 (3)	0.0518 (2)	0.0278 (11)	
H11	0.2259	0.014	0.0087	0.033*	
C12	0.3027 (4)	0.1131 (3)	0.0648 (2)	0.0248 (10)	
H12	0.3041	0.1581	0.0307	0.03*	
C13	0.2975 (3)	0.3308 (3)	0.14371 (19)	0.0171 (9)	
C14	0.1927 (3)	0.3134 (3)	0.1176 (2)	0.0250 (10)	
H14	0.1743	0.254	0.1009	0.03*	
C15	0.1145 (4)	0.3834 (3)	0.1160 (2)	0.0330 (12)	
H15	0.0427	0.3715	0.0985	0.04*	
C16	0.1417 (4)	0.4701 (3)	0.1400 (2)	0.0359 (13)	
H16	0.0882	0.5174	0.1392	0.043*	
C17	0.2453 (4)	0.4882 (3)	0.1649 (2)	0.0346 (12)	
H17	0.2639	0.5481	0.1804	0.041*	
C18	0.3229 (4)	0.4186 (3)	0.1673 (2)	0.0253 (11)	
H18	0.3943	0.431	0.1854	0.03*	
C19	0.0326 (8)	0.1586 (7)	0.2592 (6)	0.035 (3)	0.5
H19	0.093 (8)	0.172 (7)	0.280 (5)	0.04 (3)*	0.5

### Atomic displacement parameters (Å^2^)

**Table:**

	*U*^11^	*U*^22^	*U*^33^	*U*^12^	*U*^13^	*U*^23^
Pt1	0.01756 (11)	0.01119 (10)	0.01270 (11)	0	0.00113 (8)	0
Br1	0.0228 (2)	0.0222 (2)	0.0209 (2)	−0.0013 (2)	0.00405 (17)	−0.0001 (2)
Cl1	0.0518 (8)	0.0557 (8)	0.0375 (8)	−0.0015 (7)	0.0168 (7)	−0.0071 (7)
Cl2	0.064 (3)	0.0486 (16)	0.068 (4)	0.0134 (16)	0.031 (2)	0.0093 (17)
P1	0.0188 (5)	0.0119 (4)	0.0148 (5)	0.0016 (5)	0.0016 (4)	0.0005 (5)
C1	0.017 (2)	0.020 (2)	0.013 (2)	−0.0013 (16)	−0.0015 (17)	0.0012 (16)
C2	0.033 (3)	0.026 (2)	0.024 (3)	0.005 (2)	0.006 (2)	0.004 (2)
C3	0.034 (3)	0.039 (3)	0.027 (3)	0.012 (2)	0.009 (2)	−0.006 (2)
C4	0.027 (3)	0.039 (3)	0.017 (2)	−0.007 (2)	0.006 (2)	0.001 (2)
C5	0.037 (3)	0.025 (2)	0.019 (2)	−0.006 (2)	0.000 (2)	0.007 (2)
C6	0.028 (2)	0.022 (2)	0.020 (2)	−0.0002 (19)	0.003 (2)	−0.0006 (19)
C7	0.020 (2)	0.0112 (19)	0.015 (2)	0.0001 (17)	0.0033 (18)	−0.0007 (17)
C8	0.022 (2)	0.016 (2)	0.016 (2)	0.0024 (18)	0.0012 (19)	0.0003 (18)
C9	0.034 (3)	0.016 (2)	0.024 (3)	−0.0021 (19)	0.011 (2)	0.0025 (19)
C10	0.027 (2)	0.020 (2)	0.027 (3)	−0.0042 (19)	0.005 (2)	−0.007 (2)
C11	0.034 (3)	0.029 (2)	0.020 (2)	−0.004 (2)	−0.002 (2)	−0.009 (2)
C12	0.035 (3)	0.017 (2)	0.022 (3)	−0.0028 (19)	0.000 (2)	0.0037 (19)
C13	0.024 (2)	0.018 (2)	0.010 (2)	0.0035 (18)	0.0066 (18)	0.0023 (17)
C14	0.027 (3)	0.026 (2)	0.022 (2)	0.0026 (19)	0.004 (2)	0.001 (2)
C15	0.024 (3)	0.048 (3)	0.028 (3)	0.012 (2)	0.005 (2)	0.015 (2)
C16	0.043 (3)	0.038 (3)	0.028 (3)	0.028 (2)	0.012 (2)	0.014 (2)
C17	0.062 (4)	0.020 (2)	0.023 (3)	0.016 (2)	0.009 (3)	0.001 (2)
C18	0.032 (3)	0.023 (2)	0.021 (3)	0.002 (2)	−0.003 (2)	0.002 (2)
C19	0.022 (7)	0.052 (6)	0.030 (7)	−0.003 (4)	−0.006 (6)	0.001 (5)

### Geometric parameters (Å, °)

**Table:**

Pt1—P1	2.3245 (9)	C8—C9	1.396 (5)
Pt1—P1^i^	2.3245 (9)	C8—H8	0.95
Pt1—Br1	2.4417 (4)	C9—C10	1.376 (6)
Pt1—Br1^i^	2.4417 (4)	C9—H9	0.95
Cl1—C19	1.708 (12)	C10—C11	1.392 (6)
Cl1—C19^ii^	1.807 (11)	C10—H10	0.95
Cl2—Cl2^ii^	0.796 (6)	C11—C12	1.390 (6)
Cl2—C19^ii^	1.569 (11)	C11—H11	0.95
Cl2—C19	1.763 (10)	C12—H12	0.95
P1—C13	1.820 (4)	C13—C14	1.391 (6)
P1—C7	1.825 (4)	C13—C18	1.393 (6)
P1—C1	1.831 (4)	C14—C15	1.397 (6)
C1—C6	1.389 (5)	C14—H14	0.95
C1—C2	1.394 (6)	C15—C16	1.386 (7)
C2—C3	1.385 (6)	C15—H15	0.95
C2—H2	0.95	C16—C17	1.371 (7)
C3—C4	1.378 (6)	C16—H16	0.95
C3—H3	0.95	C17—C18	1.389 (6)
C4—C5	1.370 (6)	C17—H17	0.95
C4—H4	0.95	C18—H18	0.95
C5—C6	1.389 (6)	C19—C19^ii^	0.865 (18)
C5—H5	0.95	C19—Cl2^ii^	1.569 (10)
C6—H6	0.95	C19—Cl1^ii^	1.807 (11)
C7—C8	1.395 (5)	C19—H19	0.86 (9)
C7—C12	1.400 (6)		
			
P1—Pt1—P1^i^	178.54 (6)	C9—C10—C11	120.0 (4)
P1—Pt1—Br1	92.30 (3)	C9—C10—H10	120
P1^i^—Pt1—Br1	87.69 (3)	C11—C10—H10	120
P1—Pt1—Br1^i^	87.69 (3)	C12—C11—C10	120.1 (4)
P1^i^—Pt1—Br1^i^	92.30 (3)	C12—C11—H11	119.9
Br1—Pt1—Br1^i^	178.96 (3)	C10—C11—H11	119.9
Cl2^ii^—Cl2—C19^ii^	90.3 (4)	C11—C12—C7	120.1 (4)
Cl2^ii^—Cl2—C19	62.9 (3)	C11—C12—H12	119.9
C13—P1—C7	108.31 (19)	C7—C12—H12	119.9
C13—P1—C1	103.16 (18)	C14—C13—C18	119.1 (4)
C7—P1—C1	102.66 (17)	C14—C13—P1	123.9 (3)
C13—P1—Pt1	111.00 (13)	C18—C13—P1	117.0 (3)
C7—P1—Pt1	111.91 (13)	C13—C14—C15	119.9 (4)
C1—P1—Pt1	118.91 (13)	C13—C14—H14	120.1
C6—C1—C2	117.8 (4)	C15—C14—H14	120.1
C6—C1—P1	122.5 (3)	C16—C15—C14	120.0 (4)
C2—C1—P1	119.6 (3)	C16—C15—H15	120
C3—C2—C1	120.8 (4)	C14—C15—H15	120
C3—C2—H2	119.6	C17—C16—C15	120.5 (4)
C1—C2—H2	119.6	C17—C16—H16	119.8
C4—C3—C2	120.3 (4)	C15—C16—H16	119.8
C4—C3—H3	119.9	C16—C17—C18	119.7 (4)
C2—C3—H3	119.9	C16—C17—H17	120.1
C5—C4—C3	119.9 (4)	C18—C17—H17	120.1
C5—C4—H4	120.1	C17—C18—C13	120.8 (4)
C3—C4—H4	120.1	C17—C18—H18	119.6
C4—C5—C6	120.1 (4)	C13—C18—H18	119.6
C4—C5—H5	120	C19^ii^—C19—Cl2^ii^	87.8 (4)
C6—C5—H5	120	C19^ii^—C19—Cl1	82.2 (13)
C5—C6—C1	121.1 (4)	Cl2^ii^—C19—Cl1	126.8 (7)
C5—C6—H6	119.4	C19^ii^—C19—Cl2	62.8 (3)
C1—C6—H6	119.4	Cl1—C19—Cl2	110.2 (6)
C8—C7—C12	119.2 (4)	C19^ii^—C19—Cl1^ii^	69.5 (12)
C8—C7—P1	119.8 (3)	Cl2^ii^—C19—Cl1^ii^	114.8 (7)
C12—C7—P1	121.0 (3)	Cl1—C19—Cl1^ii^	110.0 (5)
C7—C8—C9	120.1 (4)	Cl2—C19—Cl1^ii^	110.3 (6)
C7—C8—H8	120	C19^ii^—C19—H19	166 (7)
C9—C8—H8	120	Cl2^ii^—C19—H19	78 (7)
C10—C9—C8	120.4 (4)	Cl1—C19—H19	109 (7)
C10—C9—H9	119.8	Cl2—C19—H19	104 (7)
C8—C9—H9	119.8	Cl1^ii^—C19—H19	113 (7)

Symmetry codes: (i) −*x*+1, *y*, −*z*+1/2; (ii) −*x*, *y*, −*z*+1/2.
